# Crystal structure of 3,6,6-trimethyl-4-oxo-1-(pyridin-2-yl)-4,5,6,7-tetra­hydro-1*H*-indazol-7-aminium chloride and its monohydrate

**DOI:** 10.1107/S205698901701667X

**Published:** 2017-11-24

**Authors:** Anatoly Mishnev, Alvis Mengots, Māris Turks

**Affiliations:** a Latvian Institute of Organic Synthesis, Aizkraukles Str. 21, Riga LV-1006, Latvia; b Institute of Technology of Organic Chemistry, Faculty of Materials Science and Applied Chemistry, Riga Technical University, P. Valdena Str. 3, Riga LV-1048, Latvia

**Keywords:** crystal structure, tetra­hydro­indazole, tetra­hydro­indazolone, hydro­chloride, hydrate

## Abstract

The title compounds, C_15_H_19_N_4_O^+^·Cl^−^ and C_15_H_19_N_4_O^+^·Cl^−^·H_2_O, obtained in attempts to synthesize metal complexes using tetra­hydro­indazole as a ligand, were characterized by NMR, IR and X-ray diffraction techniques. The partially saturated ring in the tetra­hydro­indazole core adopts a sofa conformation. An intra­molecular N—H⋯N hydrogen bond formed by the protonated amino group and the N atom of the pyridyl substituent is found in the first structure. In the hydro­chloride, the organic moieties are linked by two N—H⋯Cl^−^ hydrogen bonds, forming a *C*(4) graph-set. In the hydrate crystal, a Cl^−^ anion and a water mol­ecule assemble the moieties into infinite bands showing hydrogen-bond patterns with graph sets *C*(6), *R*_6_^4^(12) and *R*_4_^2^(8). Organic moieties form π–π stacked supra­molecular structures running along the *b* axis in both structures.

## Abstract 2

In the title compounds **1** and **2**, the organic moieties adopt flattened conformations stabilized by an intra­molecular N—H⋯N hydrogen bonds formed by the protonated amino group and the N atom of the pyridyl substituent. In **1**, the organic moieties are linked with two N—H⋯Cl^−^-type hydrogen bonds, forming a *C*(4) graph-set. In its monohydrate, **2**, the Cl^−^ anion and a water mol­ecule assemble the moieties into infinite bands showing hydrogen-bond patterns with graph sets *C*(6), 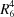
(12) and 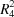
(8). Both crystals display π–π stacked supra­molecular structures running along the *b* axis.

## Chemical context   

Tetra­hydro­indazoles can be regarded as annulated pyrazole analogs (Ansari *et al.*, 2017 ▸) or as partially saturated indazoles (Gaikwad *et al.*, 2015 ▸). In either of these categories they play an important role in medicinal chemistry. Tetra­hydro­indazoles are reported to be peripherally selective cannabinoid-1 receptor inverse agonists (Matthews *et al.*, 2016 ▸), sigma-2 receptor ligands(Wu *et al.*, 2015 ▸), and inter­leukin-2 inducible T-cell kinase inhibitors (Burch *et al.*, 2015 ▸; Heifetz *et al.*, 2016 ▸). Heterocyclic compounds containing a tetra­hydro­indazole core have been researched as anti­viral agents (Bassyouni *et al.*, 2016 ▸) and compounds with anti­oxidant properties (Polo *et al.*, 2016 ▸). With appropriate side-chain decorations, they also possess COX-2 inhibitory activity (Abdel-Rahman *et al.*, 2012 ▸) and can inhibit bacterial type II topoisomerases (Wiener *et al.*, 2007 ▸). The latter has led to the development of compounds with both anti­tumor and anti­microbial activity (Faidallah *et al.*, 2013 ▸), including novel anti­tuberculosis agents (Guo *et al.*, 2010 ▸).
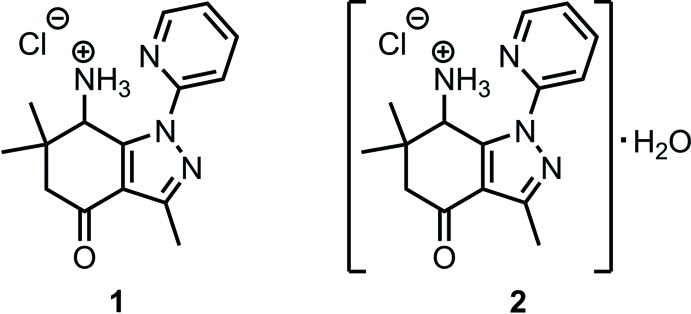


The broad application spectrum of tetra­hydro­indazoles has led to the development of synthetic methodologies. Thus, traditional approaches using a combination of either α,β-unsaturated ketones (Nakhai & Bergman, 2009 ▸) or dicarbonyl compounds (Murugavel *et al.*, 2010 ▸), or tricarbonyl compounds (Kim *et al.*, 2010 ▸; Scala *et al.*, 2015 ▸) with hydrazines have been significantly updated and improved. In addition, the microwave-assisted synthesis of tetra­hydro­indazoles has been reported (Silva *et al.*, 2006 ▸; Polo *et al.*, 2016 ▸). It is inter­esting to note that compounds possessing free NH-functionality in the pyrazole ring have been studied thoroughly for their tautomeric equilibria (Claramunt *et al.*, 2006 ▸). Additionally, tetra­hydro­indazolones substituted with 2-amino­benzamides have been studied as fluorescent probes (Jia *et al.*, 2012 ▸). Other studies on side-chain modifications include the synthesis of polyfluoro­alkyl-substituted analogs (Khlebnikova *et al.*, 2012 ▸), triazole-functionalized tetra­hydro­indazolones (Strakova *et al.*, 2009 ▸) and their conjugation with biologically active natural products such as lupane triterpenoids (Khlebnicova *et al.*, 2017 ▸). Among other synthetic approaches, the Ritter reaction provides a fast entry into structural modifications and is applicable to obtain a combinatorial library of compounds (Turks *et al.*, 2012 ▸). Combinatorial chemistry methodology has been reported for the construction of tetra­hydro­indazolones in enanti­omerically pure pairs (Song *et al.*, 2012 ▸). Also, enanti­omerically pure 7-amino-tetra­hydro­indazolones (Strakova *et al.*, 2011 ▸) have been obtained. For these reasons, we were inter­ested in the synthesis of 7-amino-3,6,6-trimethyl-1-(pyridin-2-yl)-1,5,6,7-tetra­hydro-4*H*-indazol-4-one for use as a starting material for further structural modifications. Herein, the structures of the corresponding hydro­chloride **1** and its hydrate **2** are reported.

## Structural commentary   

**Figure 1:**
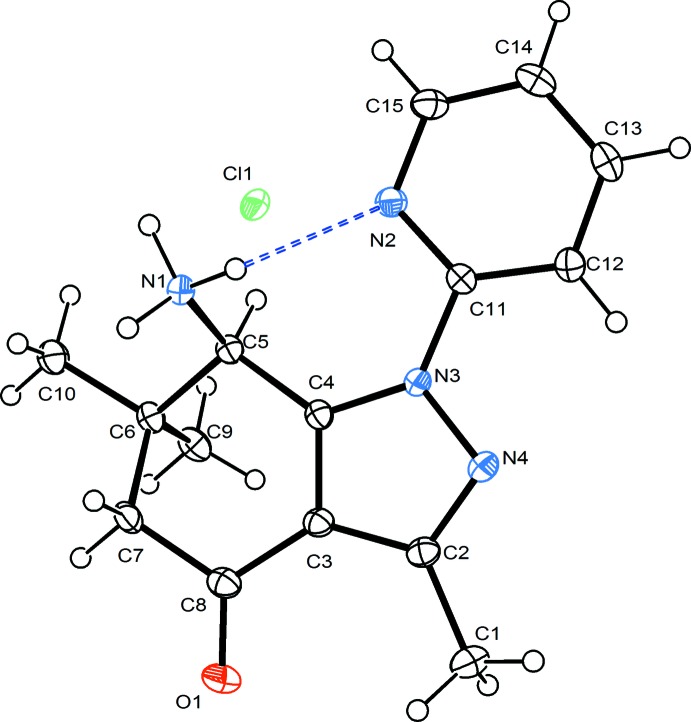
*ORTEP* view of the asymmetric unit of **1** showing the atom-numbering scheme and 50% probability displacement ellipsoids. The intra­molecular hydrogen bond is shown with dashed lines.

Figs. 1 ▸ and 2 ▸ show the asymmetric units of the hydro­chloride (**1**) and its hydrate (**2**) with the symmetry-independent hydrogen bonds. The geometry and conformation of the organic cation in compounds **1** and **2** are substanti­ally similar. The pyrazole ring is planar within an r.m.s. deviation of the fitted atoms of 0.0059 Å in **1** and 0.0092 Å in **2**. In both structures, the partially saturated ring adopts a sofa conformation. The distance of atom C6 from the mean plane formed by atoms C3–C5/C7/C8 (r.m.s. deviation of fitted atoms = 0.0495 Å in **1** and 0.0558 Å in **2**) is 0.639 (2) Å in **1** and 0.642 (2) Å in **2**. The dihedral angle between the latter plane and pyrazole ring is 5.79 (6)° in **1** and 6.48 (4)° in **2**. On the other hand, the dihedral angle between the pyrazole ring and its pyridyl substituent is 11.91 (6)° [torsion angle N4—N3—C11—C12 = 10.7 (2)°] in **1** and 7.22 (5)° [torsion angle N4—N3—C11—C12 = 4.6 (2)°] in **2**. An intra­molecular N—H⋯N hydrogen bond formed by the protonated amino group and nitro­gen atom of pyridyl substituent is found in **1** (Table 1 ▸).

## Supra­molecular features   

**Figure 3:**
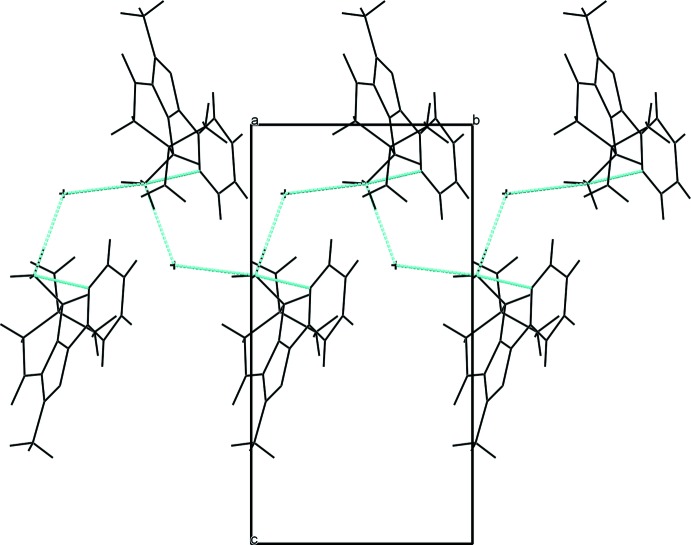
The crystal packing of compound **1**, viewed along the *a* axis. The hydrogen bonds are shown as dashed lines (see Table 1 ▸).

**Figure 4:**
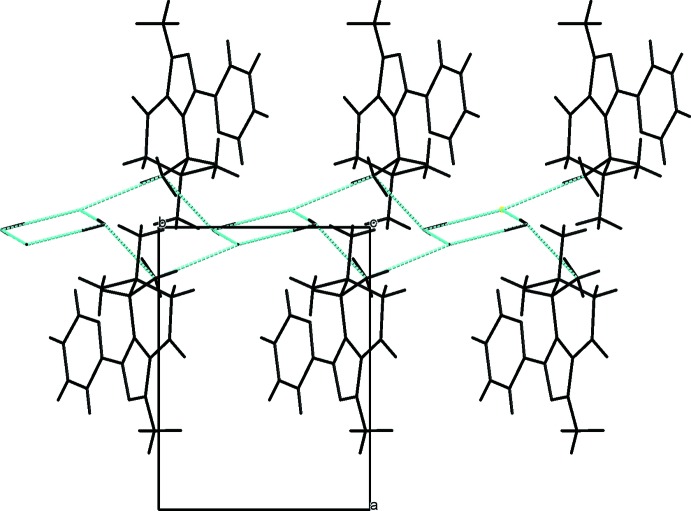
The crystal packing of compound **2**, viewed along the *c* axis. The hydrogen bonds are shown as dashed lines (see Table 2 ▸).

In the crystal of compound **1**, the organic moieties are linked by two types of N—H⋯Cl^−^ hydrogen bonds into infinite chains along the *b*-axis direction (Table 1 ▸). According to Etter (1990 ▸), the hydrogen-bond pattern in **1** can be described by a *C*(4) graph set. The packing of **1** is shown in Fig. 3 ▸. In the structure of **2**, in addition to participating in an intra­molecular hydrogen bond, the protonated amino group also forms two inter­molecular hydrogen bonds with the Cl^−^ anion and a water mol­ecule (Table 2 ▸). Each Cl^−^ anion and water mol­ecule takes part in three inter­molecular hydrogen bonds. The organic cations are bridged by a pair of Cl^−^ anions and a water mol­ecule, thus assembling the moieties into infinite bands running along the *b-*axis direction. The hydrogen-bond pattern can be described by graph sets *C*(6), 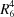
(12) and 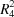
(8). The packing of **2** is shown in Fig. 4 ▸.

**Figure 5:**
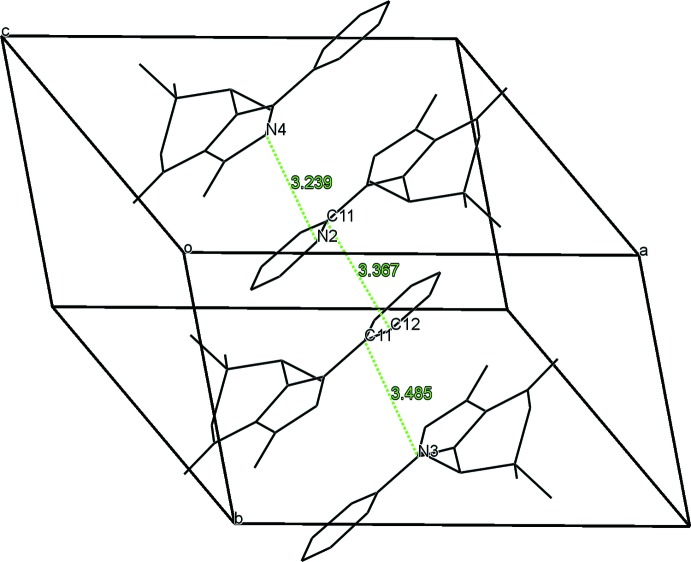
View of stacks of organic moieties in the crystal structure of **1**. H atoms and chloride anions are not shown for clarity.

**Figure 6:**
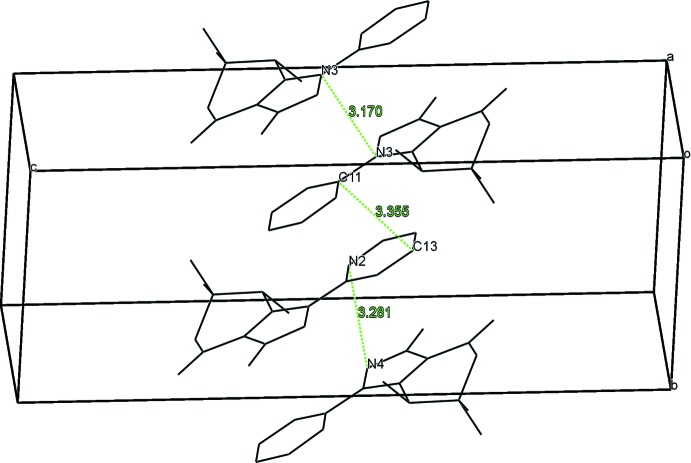
View of stacks of organic moieties in the crystal structure of **2**. H atoms, chloride anions and water mol­ecules are not shown for clarity.

In the crystal of **1**, the organic moieties form stacks running along the *b* axis which are stabilized by π–π inter­actions (Fig. 5 ▸). The distance between the centroids of the pyridine and pyrazole rings of adjacent mol­ecules is 3.585 (2) Å. The shortest contact is 3.239 (2) Å between atoms N2 and N4 of two inversion-related mol­ecules (Fig. 5 ▸). In the crystal of **2**, the organic moieties also form π–π-stacked supra­molecular structures running along the *b-*axis direction (Fig. 6 ▸). The distance between the centroids of the pyridine rings of adjacent mol­ecules is 3.748 (2) Å. The shortest contact is 3.170 (2) Å between the N3 atoms of two inversion-related mol­ecules (Fig. 6 ▸).

## Database survey   

A search of the Cambridge Structural Database (Version 5.38; Groom *et al.*, 2016 ▸) for the 3,6,6-trimethyl-4-oxo-4,5,6,7-tetra­hydro-1*H*-indazole core revealed five structurally close compounds: UXAQUG, UXARAN, UXARER, UXARIV, UXAROB (Strakova *et al.*, 2011 ▸). These compounds differ from compounds **1** and **2** by the substituents at the positions of atoms N3 and C5. In all examples, the partially saturated ring in the indazole fragment adopts a sofa conformation. However, the phenyl ring at the position N3 forms much larger dihedral angles with the pyrazole ring than with the pyridyl substituent in the structures reported here.

## Synthesis and crystallization   

The synthesis of the title compounds is depicted in the reaction scheme below. The 7-amino­tetra­hydro­indazolone derivative **4** was prepared by an analogy of the procedure published by Strakova *et al.* (2011 ▸) from the known precursor **3** (Strakova *et al.*, 2009 ▸). In our attempts to synthesize metal complexes with ligand **4**, we obtained the hydro­chloride salt **1** in its anhydrous form. It can be explained by the acidity of cobalt chloride hexa­hydrate, which was used in the selected experiment. This prompted us to develop a preparative synthesis of the hydro­chloride salt. This was achieved by the formation and precipitation of crude hydro­chloride in ethyl acetate solution. Its crystallization from water provided the hydro­chloride hydrate **2**.
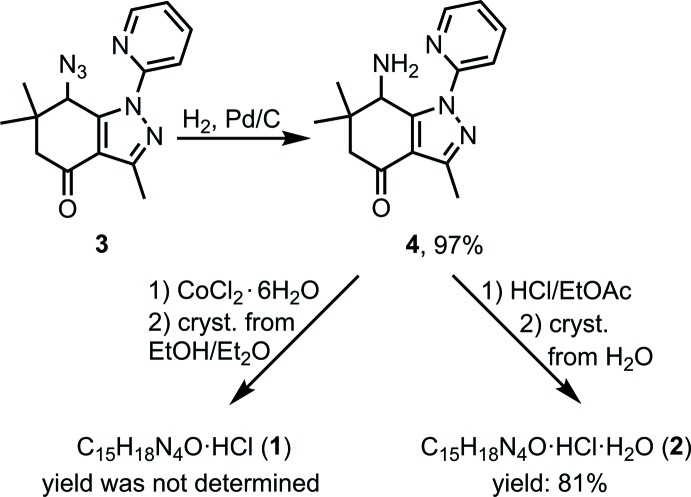


**7-Amino-3,6,6-trimethyl-1-(pyridin-2-yl)-1,5,6,7-tetra­hydro-4** ***H*** **-indazol-4-one (4):** Gaseous H_2_ was bubbled for 10 min. through a solution/suspension of compound **3** (0.80 g, 2.7 mmol) and 10% Pd/C (80 mg) in a mixture of EtOH (10 mL) and THF (2 mL). The resulting reaction mixture was stirred under an H_2_ atmosphere at standard temperature and pressure for 3 h (TLC control). The catalyst was filtered through a celite pad and the filtrate was evaporated to dryness. The resulting amorphous solid was dried under reduced pressure to yield amine **4** (0.71 g, 97%) as a colorless powder. M.p. 390–392 K; *R*_f_ = 0.14 (Hex:EtOAc:Et_3_N = 8:1:0.5). IR (KBr), υ (cm^−1^): 3360, 3295, 3055, 2985, 2955, 2945, 2930, 2890, 2865, 1670, 1590, 1575, 1540, 1465, 1455, 1285, 1250, 1145, 1085, 1075, 1035, 995. ^1^H NMR (CDCl_3_, 300 MHz) δ (ppm): 8.48 [*m*, 1H, H-C(Py)], 7.99 [*d*, *J* = 8.3 Hz, 1H, H-C(Py)], 7.87 [*m*, 1H, H-C(Py)], 7.26 [*m*, 1H, H-C(Py)], 4.27 (*s*, 1H, H-C7), 2.82 (*d*, *J* = 16.8 Hz, 1H, H_a_-C5), 2.54 (*s*, 3H, H_3_C-C3), 2.18 (*d*, *J* = 16.8 Hz, 1H, H_b_-C5), 2.08 (*bs*, 2H, H_2_N-C7) 1.26, 1.02 (2*s*, 6H, H_3_C-C6).^13^C NMR (75.5 MHz, CDCl_3_), δ (ppm): 194.1, 153.9, 152.4, 150.4, 148.0, 139.1, 122.1, 116.5, 115.9, 53.8, 47.8, 38.4, 27.3, 26.6, 13.7. Analysis calculated: (C_15_H_18_N_4_O) C, 66.64; H, 6.71; N, 20.73. Found: C, 66.56; H, 6.68; N, 20.74.

**3,6,6-Trimethyl-4-oxo-1-(pyridin-2-yl)-4,5,6,7-tetra­hydro-1** ***H*** **-indazol-7-aminium chloride (1):** A solution of CoCl_2_·6H_2_O (24 mg, 0.1 mmol) in ethanol (2 mL) was added to a solution of amine **4** (27 mg, 0.1 mmol) in ethanol (2 mL). The resulting reaction mixture was maturated at ambient temperature for 24 h. Then a part of it (1.2 mL) was transferred into an NMR tube and Et_2_O (0.8 mL) was added carefully on the top of the ethanol solution. After two days, colorless crystals of **1** were collected form the wall of the NMR tube. The product was characterized spectroscopically in its hydrate form (see below).

**3,6,6-Trimethyl-4-oxo-1-(pyridin-2-yl)-4,5,6,7-tetra­hydro-1** ***H*** **-indazol-7-aminium chloride hydrate (2):** A solution of HCl in EtOAc (0.5 *M*, 1.48 mL, 0.74 mmol, 1.0 equiv.) was added to a solution of amine **4** (0.20 g, 0.74 mmol, 1.0 equiv.) in EtOAc (2 mL) at ambient temperature. The resulting precipitate was filtered and washed on the filter with DCM. The the crude product was crystallized from water to obtain colorless crystals of **2** (195 mg, 81%) suitable for X-ray analysis. M.p. 543 K (decomp.); IR (KBr), υ (cm^−1^): 3430 (*br.s*), 3145, 3100, 3035, 2965, 2880, 2750, 2575, 1955 (*br.s*), 1685, 1600, 1545, 1520, 1490, 1465, 1450, 1400, 1375, 1360, 1295, 1245, 1140, 1045, 1000, 955. ^1^H NMR (300MHz, D_2_O), δ (ppm): δ 8.55 [*m*, 1H, H-C(Py)], 8.12 [*m*,1H,H-C (Py)], 7.90 [*d*, *J* = 8.3 Hz, 1H, H-C(Py)], 7.53 [*m*, 1H, H-C(Py)], 4.84 (*s*, 1H, H-C7), 3.00 (*d*, *J* =17.8 Hz, 1H, H_a_-C5), 2.54 (*s*, 3H, H_3_C-C3), 2.45 (*d*, *J* = 17.8Hz,1H,H_b_-C5), 1.36, 1.10 (2*s*, 6H, H_3_C-C6). ^13^C NMR (75.5 MHz, DMSO-*d*_6_), δ (ppm):192.3, 151.4, 149.0, 148.0, 144.4, 140.1, 122.8, 118.0, 114.6, 51.4, 47.1, 37.2, 26.8, 25.4, 13.2. Analysis calculated: (C_15_H_18_N_4_O·HCl·H_2_O) C, 55.47; H, 6.52; N, 17.25. Found: C, 55.78; H,6.40; N, 17.29.

## Refinement   

Crystal data, data collection and structure refinement details are summarized in Table 3 ▸. Hydrogen atoms bonded to heteroatoms were refined isotropically. Other H atoms were included in the refinement at geometrically calculated positions with C—H = 0.95–0.99Å and treated as riding with *U*_iso_(H) = 1.2*U*_eq_(C) or 1.5*U*_eq_(C-methyl).

## Supplementary Material

Crystal structure: contains datablock(s) 1, 2, global. DOI: 10.1107/S205698901701667X/eb2002sup1.cif

Structure factors: contains datablock(s) 1. DOI: 10.1107/S205698901701667X/eb20021sup2.hkl

Supporting information file. DOI: 10.1107/S205698901701667X/eb20021sup4.mol

Structure factors: contains datablock(s) 2. DOI: 10.1107/S205698901701667X/eb20022sup3.hkl

Supporting information file. DOI: 10.1107/S205698901701667X/eb20022sup5.mol

Supporting information file. DOI: 10.1107/S205698901701667X/eb20021sup6.cml

Supporting information file. DOI: 10.1107/S205698901701667X/eb20022sup7.cml

CCDC references: 1586488, 1586487

Additional supporting information:  crystallographic information; 3D view; checkCIF report

## Figures and Tables

**Figure 2:**
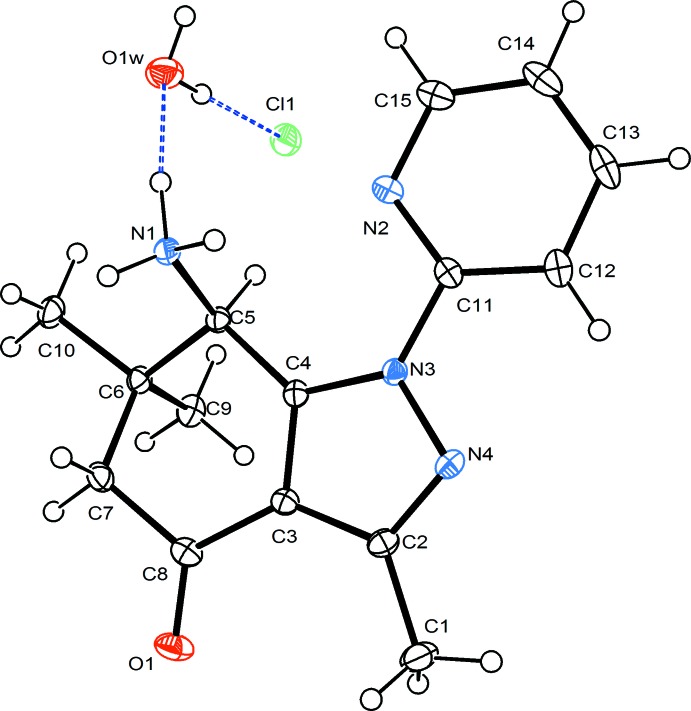
*ORTEP* view of the asymmetric unit of **2** showing the atom-numbering scheme and 50% probability displacement ellipsoids. The intra­molecular hydrogen bonds are shown with dashed lines.

**Table 1:** Hydrogen-bond geometry (Å, °) for (1)

*D*—H⋯*A*	*D*—H	H⋯*A*	*D*⋯*A*	*D*—H⋯*A*
N1—H1*N*1⋯N2	0.97 (2)	2.42 (2)	2.928 (2)	112 (2)
N1—H2*N*1⋯Cl1^i^	0.97 (2)	2.08 (2)	3.034 (2)	168 (2)
N1—H3*N*1⋯Cl1^ii^	0.93 (2)	2.27 (2)	3.188 (2)	167 (2)

Symmetry codes: (i) 
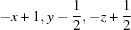
; (ii) 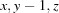.

**Table 2:** Hydrogen-bond geometry (Å, °) for (2)

*D*—H⋯*A*	*D*—H	H⋯*A*	*D*⋯*A*	*D*—H⋯*A*
O1*W*—H1*W*⋯Cl1^i^	0.80 (3)	2.39 (3)	3.185 (2)	176 (3)
O1*W*—H2*W*⋯Cl1	0.94 (3)	2.31 (3)	3.247 (2)	179 (2)
N1—H1*N*1⋯Cl1^ii^	0.85 (2)	2.40 (2)	3.228 (2)	165 (2)
N1—H3*N*1⋯O1*W*	0.95 (2)	1.85 (3)	2.775 (2)	162 (2)

Symmetry codes: (i) 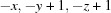; (ii) 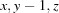.

**Table 3:** Experimental details

	(1)	(2)
Crystal data		
Chemical formula	C_15_H_19_N_4_O^+^·Cl^−^	C_15_H_19_N_4_O^+^·Cl^−^·H_2_O
*M*_r_	306.79	324.81
Crystal system, space group	Monoclinic, *P*2_1_/*c*	Monoclinic, *P*2_1_/*c*
Temperature (K)	190	190
*a*, *b*, *c* (Å)	13.5411 (4), 7.7421 (2), 19.2457 (5)	10.1855 (2), 7.4951 (2), 20.7961 (4)
β (°)	130.493 (2)	100.545 (1)
*V* (Å^3^)	1534.39 (8)	1560.79 (6)
*Z*	4	4
Radiation type	Mo *K*α	Mo *K*α
μ (mm^−1^)	0.25	0.26
Crystal size (mm)	0.38 × 0.32 × 0.15	0.42 × 0.25 × 0.14

Data collection		
Diffractometer	Nonius KappaCCD	Nonius KappaCCD
No. of measured, independent and observed [*I* > 2σ(*I*)] reflections	5860, 3486, 2715	5549, 3552, 2874
*R*_int_	0.027	0.023
(sin θ/λ)_max_ (Å^−1^)	0.649	0.654

Refinement		
*R*[*F* ^2^ > 2σ(*F* ^2^)], *wR*(*F* ^2^), *S*	0.043, 0.101, 1.03	0.039, 0.102, 1.06
No. of reflections	3486	3552
No. of parameters	205	222
H-atom treatment	H atoms treated by a mixture of independent and constrained refinement	H atoms treated by a mixture of independent and constrained refinement
Δρ_max_, Δρ_min_ (e Å^−3^)	0.29, −0.25	0.29, −0.28

Computer programs: *COLLECT* (Bruker, 2004 ▸), *SCALEPACK* (Otwinowski & Minor, 1997 ▸), *DENZO* (Otwinowski & Minor, 1997 ▸), *SIR2004* (Burla *et al.*, 2005 ▸), *SHELXL2017* (Sheldrick, 2015 ▸), *ORTEP-3 for Windows* (Farrugia, 2012 ▸) and *publCIF* (Westrip, 2010 ▸).

## supplementary crystallographic information

### 3,6,6-Trimethyl-4-oxo-1-(pyridin-2-yl)-4,5,6,7-tetrahydro-1H-indazol-7-aminium chloride (1) Crystal data

**Table:**

C_15_H_19_N_4_O^+^·Cl^−^	*F*(000) = 648
*M**_r_* = 306.79	*D*_x_ = 1.328 Mg m^−^^3^
Monoclinic, *P*2_1_/*c*	Mo *K*α radiation, λ = 0.71073 Å
*a* = 13.5411 (4) Å	Cell parameters from 6801 reflections
*b* = 7.7421 (2) Å	θ = 1.0–27.5°
*c* = 19.2457 (5) Å	µ = 0.25 mm^−^^1^
β = 130.493 (2)°	*T* = 190 K
*V* = 1534.39 (8) Å^3^	Block, colourless
*Z* = 4	0.38 × 0.32 × 0.15 mm

### 3,6,6-Trimethyl-4-oxo-1-(pyridin-2-yl)-4,5,6,7-tetrahydro-1H-indazol-7-aminium chloride (1) Data collection

**Table:**

Nonius KappaCCD diffractometer	*R*_int_ = 0.027
Radiation source: fine-focus sealed tube	θ_max_ = 27.5°, θ_min_ = 3.0°
CCD scans	*h* = −17→17
5860 measured reflections	*k* = −10→9
3486 independent reflections	*l* = −24→24
2715 reflections with *I* > 2σ(*I*)	

### 3,6,6-Trimethyl-4-oxo-1-(pyridin-2-yl)-4,5,6,7-tetrahydro-1H-indazol-7-aminium chloride (1) Refinement

**Table:**

Refinement on *F*^2^	0 restraints
Least-squares matrix: full	Hydrogen site location: mixed
*R*[*F*^2^ > 2σ(*F*^2^)] = 0.043	H atoms treated by a mixture of independent and constrained refinement
*wR*(*F*^2^) = 0.101	*w* = 1/[σ^2^(*F*_o_^2^) + (0.0363*P*)^2^ + 0.7495*P*] where *P* = (*F*_o_^2^ + 2*F*_c_^2^)/3
*S* = 1.03	(Δ/σ)_max_ = 0.001
3486 reflections	Δρ_max_ = 0.29 e Å^−^^3^
205 parameters	Δρ_min_ = −0.25 e Å^−^^3^

### 3,6,6-Trimethyl-4-oxo-1-(pyridin-2-yl)-4,5,6,7-tetrahydro-1H-indazol-7-aminium chloride (1) Special details

**Table:**

Geometry. All esds (except the esd in the dihedral angle between two l.s. planes) are estimated using the full covariance matrix. The cell esds are taken into account individually in the estimation of esds in distances, angles and torsion angles; correlations between esds in cell parameters are only used when they are defined by crystal symmetry. An approximate (isotropic) treatment of cell esds is used for estimating esds involving l.s. planes.

### 3,6,6-Trimethyl-4-oxo-1-(pyridin-2-yl)-4,5,6,7-tetrahydro-1H-indazol-7-aminium chloride (1) Fractional atomic coordinates and isotropic or equivalent isotropic displacement parameters (Å^2^)

**Table:**

	*x*	*y*	*z*	*U*_iso_*/*U*_eq_	
Cl1	0.63264 (4)	0.65297 (6)	0.33536 (3)	0.03342 (14)	
O1	0.98736 (12)	−0.08281 (19)	0.66919 (8)	0.0361 (3)	
N3	0.59725 (12)	0.17416 (17)	0.53425 (8)	0.0187 (3)	
N4	0.65309 (13)	0.13670 (18)	0.62325 (9)	0.0219 (3)	
C4	0.67526 (15)	0.1258 (2)	0.51633 (10)	0.0193 (3)	
N2	0.41873 (13)	0.26763 (19)	0.38981 (9)	0.0230 (3)	
C12	0.42625 (16)	0.3337 (2)	0.51532 (11)	0.0232 (4)	
H12	0.471148	0.327724	0.577688	0.028*	
C11	0.47588 (15)	0.2621 (2)	0.47773 (10)	0.0190 (3)	
N1	0.55052 (14)	0.0192 (2)	0.35950 (9)	0.0220 (3)	
C13	0.30686 (17)	0.4145 (2)	0.45593 (12)	0.0282 (4)	
H13	0.267881	0.460408	0.477604	0.034*	
C3	0.78494 (15)	0.0540 (2)	0.59559 (10)	0.0211 (3)	
C6	0.78026 (16)	0.1295 (2)	0.44709 (11)	0.0246 (4)	
C15	0.30553 (16)	0.3529 (2)	0.33478 (12)	0.0276 (4)	
H15	0.265092	0.362741	0.273282	0.033*	
C8	0.88681 (16)	−0.0231 (2)	0.59948 (11)	0.0244 (4)	
C5	0.65109 (15)	0.1485 (2)	0.42887 (10)	0.0207 (3)	
H5	0.616867	0.265008	0.405413	0.025*	
C2	0.76718 (16)	0.0664 (2)	0.66070 (11)	0.0224 (4)	
C10	0.75046 (18)	0.1108 (3)	0.35558 (12)	0.0325 (4)	
H10A	0.830139	0.113448	0.366104	0.049*	
H10B	0.695686	0.204248	0.315864	0.049*	
H10C	0.706960	0.002985	0.327613	0.049*	
C14	0.24569 (17)	0.4268 (2)	0.36430 (13)	0.0297 (4)	
H14	0.166419	0.483251	0.323640	0.036*	
C7	0.85647 (16)	−0.0288 (2)	0.50865 (11)	0.0253 (4)	
H7A	0.806434	−0.132217	0.476113	0.030*	
H7B	0.937384	−0.037310	0.520035	0.030*	
C1	0.85803 (17)	0.0141 (3)	0.75907 (11)	0.0312 (4)	
H1A	0.809647	−0.005846	0.778844	0.047*	
H1B	0.920174	0.104542	0.795200	0.047*	
H1C	0.902572	−0.089806	0.766326	0.047*	
C9	0.86206 (17)	0.2931 (2)	0.49510 (13)	0.0301 (4)	
H9A	0.815252	0.391477	0.456570	0.045*	
H9B	0.942494	0.281965	0.507027	0.045*	
H9C	0.879617	0.308100	0.551734	0.045*	
H1N1	0.487 (2)	0.000 (3)	0.3670 (14)	0.046 (6)*	
H2N1	0.503 (2)	0.062 (3)	0.2980 (16)	0.054 (7)*	
H3N1	0.584 (2)	−0.088 (3)	0.3627 (14)	0.042 (6)*	

### 3,6,6-Trimethyl-4-oxo-1-(pyridin-2-yl)-4,5,6,7-tetrahydro-1H-indazol-7-aminium chloride (1) Atomic displacement parameters (Å^2^)

**Table:**

	*U*^11^	*U*^22^	*U*^33^	*U*^12^	*U*^13^	*U*^23^
Cl1	0.0363 (3)	0.0353 (3)	0.0225 (2)	0.0052 (2)	0.01632 (19)	−0.00482 (19)
O1	0.0235 (7)	0.0442 (8)	0.0281 (6)	0.0113 (6)	0.0113 (6)	0.0038 (6)
N3	0.0190 (7)	0.0211 (7)	0.0168 (6)	0.0005 (6)	0.0119 (5)	0.0004 (6)
N4	0.0244 (7)	0.0234 (7)	0.0184 (6)	−0.0002 (6)	0.0141 (6)	0.0012 (6)
C4	0.0189 (8)	0.0186 (8)	0.0210 (7)	−0.0011 (6)	0.0132 (7)	−0.0020 (7)
N2	0.0207 (7)	0.0263 (7)	0.0216 (7)	0.0011 (6)	0.0136 (6)	0.0027 (6)
C12	0.0272 (9)	0.0200 (8)	0.0268 (8)	0.0003 (7)	0.0195 (7)	0.0005 (7)
C11	0.0189 (8)	0.0157 (8)	0.0232 (8)	−0.0015 (6)	0.0140 (7)	−0.0004 (7)
N1	0.0204 (7)	0.0266 (8)	0.0191 (7)	0.0023 (6)	0.0129 (6)	−0.0013 (6)
C13	0.0299 (9)	0.0235 (9)	0.0400 (10)	0.0021 (8)	0.0267 (8)	−0.0010 (8)
C3	0.0187 (8)	0.0209 (8)	0.0194 (7)	−0.0011 (7)	0.0105 (6)	−0.0019 (7)
C6	0.0218 (8)	0.0297 (9)	0.0264 (8)	0.0031 (7)	0.0176 (7)	0.0007 (8)
C15	0.0230 (8)	0.0299 (9)	0.0252 (8)	0.0022 (8)	0.0136 (7)	0.0063 (8)
C8	0.0203 (8)	0.0213 (8)	0.0254 (8)	−0.0005 (7)	0.0122 (7)	−0.0016 (7)
C5	0.0205 (8)	0.0220 (8)	0.0212 (7)	0.0022 (7)	0.0143 (7)	0.0010 (7)
C2	0.0214 (8)	0.0212 (8)	0.0201 (8)	−0.0022 (7)	0.0114 (7)	−0.0010 (7)
C10	0.0285 (9)	0.0455 (12)	0.0315 (9)	0.0064 (9)	0.0230 (8)	0.0043 (9)
C14	0.0224 (9)	0.0245 (9)	0.0379 (10)	0.0048 (7)	0.0176 (8)	0.0055 (8)
C7	0.0198 (8)	0.0281 (9)	0.0290 (9)	0.0042 (7)	0.0163 (7)	−0.0020 (8)
C1	0.0291 (9)	0.0364 (11)	0.0205 (8)	0.0007 (8)	0.0127 (8)	0.0025 (8)
C9	0.0251 (9)	0.0299 (10)	0.0390 (10)	0.0022 (8)	0.0224 (8)	0.0014 (8)

### 3,6,6-Trimethyl-4-oxo-1-(pyridin-2-yl)-4,5,6,7-tetrahydro-1H-indazol-7-aminium chloride (1) Geometric parameters (Å, º)

**Table:**

O1—C8	1.223 (2)	C6—C10	1.537 (2)
N3—C4	1.360 (2)	C6—C7	1.544 (2)
N3—N4	1.3811 (18)	C6—C5	1.552 (2)
N3—C11	1.424 (2)	C15—C14	1.380 (3)
N4—C2	1.325 (2)	C15—H15	0.9300
C4—C3	1.378 (2)	C8—C7	1.515 (2)
C4—C5	1.502 (2)	C5—H5	0.9800
N2—C11	1.328 (2)	C2—C1	1.496 (2)
N2—C15	1.341 (2)	C10—H10A	0.9600
C12—C13	1.383 (2)	C10—H10B	0.9600
C12—C11	1.383 (2)	C10—H10C	0.9600
C12—H12	0.9300	C14—H14	0.9300
N1—C5	1.509 (2)	C7—H7A	0.9700
N1—H1N1	0.97 (2)	C7—H7B	0.9700
N1—H2N1	0.97 (2)	C1—H1A	0.9600
N1—H3N1	0.93 (2)	C1—H1B	0.9600
C13—C14	1.381 (3)	C1—H1C	0.9600
C13—H13	0.9300	C9—H9A	0.9600
C3—C2	1.424 (2)	C9—H9B	0.9600
C3—C8	1.459 (2)	C9—H9C	0.9600
C6—C9	1.535 (3)		
			
C4—N3—N4	111.53 (12)	C3—C8—C7	114.43 (14)
C4—N3—C11	129.76 (13)	C4—C5—N1	109.18 (13)
N4—N3—C11	118.61 (12)	C4—C5—C6	110.02 (13)
C2—N4—N3	105.47 (13)	N1—C5—C6	111.99 (13)
N3—C4—C3	106.66 (13)	C4—C5—H5	108.5
N3—C4—C5	127.76 (14)	N1—C5—H5	108.5
C3—C4—C5	125.56 (14)	C6—C5—H5	108.5
C11—N2—C15	116.33 (14)	N4—C2—C3	110.63 (14)
C13—C12—C11	116.96 (15)	N4—C2—C1	120.64 (15)
C13—C12—H12	121.5	C3—C2—C1	128.72 (16)
C11—C12—H12	121.5	C6—C10—H10A	109.5
N2—C11—C12	125.07 (15)	C6—C10—H10B	109.5
N2—C11—N3	114.67 (13)	H10A—C10—H10B	109.5
C12—C11—N3	120.26 (14)	C6—C10—H10C	109.5
C5—N1—H1N1	110.3 (13)	H10A—C10—H10C	109.5
C5—N1—H2N1	110.9 (14)	H10B—C10—H10C	109.5
H1N1—N1—H2N1	106.3 (18)	C15—C14—C13	118.12 (16)
C5—N1—H3N1	114.2 (13)	C15—C14—H14	120.9
H1N1—N1—H3N1	107.5 (19)	C13—C14—H14	120.9
H2N1—N1—H3N1	107.3 (19)	C8—C7—C6	114.06 (14)
C14—C13—C12	119.76 (16)	C8—C7—H7A	108.7
C14—C13—H13	120.1	C6—C7—H7A	108.7
C12—C13—H13	120.1	C8—C7—H7B	108.7
C4—C3—C2	105.68 (14)	C6—C7—H7B	108.7
C4—C3—C8	121.68 (14)	H7A—C7—H7B	107.6
C2—C3—C8	132.55 (15)	C2—C1—H1A	109.5
C9—C6—C10	108.51 (15)	C2—C1—H1B	109.5
C9—C6—C7	109.37 (14)	H1A—C1—H1B	109.5
C10—C6—C7	110.59 (14)	C2—C1—H1C	109.5
C9—C6—C5	108.97 (14)	H1A—C1—H1C	109.5
C10—C6—C5	109.35 (13)	H1B—C1—H1C	109.5
C7—C6—C5	110.02 (14)	C6—C9—H9A	109.5
N2—C15—C14	123.68 (16)	C6—C9—H9B	109.5
N2—C15—H15	118.2	H9A—C9—H9B	109.5
C14—C15—H15	118.2	C6—C9—H9C	109.5
O1—C8—C3	123.57 (16)	H9A—C9—H9C	109.5
O1—C8—C7	121.97 (15)	H9B—C9—H9C	109.5
			
C4—N3—N4—C2	0.69 (18)	N3—C4—C5—N1	−74.7 (2)
C11—N3—N4—C2	−176.00 (14)	C3—C4—C5—N1	107.05 (18)
N4—N3—C4—C3	0.27 (18)	N3—C4—C5—C6	162.06 (16)
C11—N3—C4—C3	176.49 (15)	C3—C4—C5—C6	−16.2 (2)
N4—N3—C4—C5	−178.26 (15)	C9—C6—C5—C4	−74.59 (17)
C11—N3—C4—C5	−2.0 (3)	C10—C6—C5—C4	166.95 (14)
C15—N2—C11—C12	1.4 (2)	C7—C6—C5—C4	45.31 (18)
C15—N2—C11—N3	−178.55 (14)	C9—C6—C5—N1	163.80 (14)
C13—C12—C11—N2	1.1 (3)	C10—C6—C5—N1	45.35 (19)
C13—C12—C11—N3	−178.92 (15)	C7—C6—C5—N1	−76.29 (16)
C4—N3—C11—N2	14.7 (2)	N3—N4—C2—C3	−1.36 (18)
N4—N3—C11—N2	−169.29 (14)	N3—N4—C2—C1	177.90 (15)
C4—N3—C11—C12	−165.27 (16)	C4—C3—C2—N4	1.55 (19)
N4—N3—C11—C12	10.7 (2)	C8—C3—C2—N4	−174.86 (17)
C11—C12—C13—C14	−2.6 (3)	C4—C3—C2—C1	−177.64 (17)
N3—C4—C3—C2	−1.05 (18)	C8—C3—C2—C1	6.0 (3)
C5—C4—C3—C2	177.52 (15)	N2—C15—C14—C13	1.0 (3)
N3—C4—C3—C8	175.84 (15)	C12—C13—C14—C15	1.7 (3)
C5—C4—C3—C8	−5.6 (3)	O1—C8—C7—C6	−145.62 (17)
C11—N2—C15—C14	−2.5 (3)	C3—C8—C7—C6	36.1 (2)
C4—C3—C8—O1	177.45 (17)	C9—C6—C7—C8	62.12 (18)
C2—C3—C8—O1	−6.6 (3)	C10—C6—C7—C8	−178.44 (14)
C4—C3—C8—C7	−4.4 (2)	C5—C6—C7—C8	−57.54 (19)
C2—C3—C8—C7	171.59 (17)		

### 3,6,6-Trimethyl-4-oxo-1-(pyridin-2-yl)-4,5,6,7-tetrahydro-1H-indazol-7-aminium chloride (1) Hydrogen-bond geometry (Å, º)

**Table:**

*D*—H···*A*	*D*—H	H···*A*	*D*···*A*	*D*—H···*A*
C12—H12···Cl1^i^	0.93	2.80	3.4470 (17)	127
C14—H14···O1^ii^	0.93	2.44	3.277 (2)	150
C7—H7*A*···Cl1^iii^	0.97	2.71	3.6401 (18)	160
C1—H1*B*···O1^iv^	0.96	2.60	3.503 (2)	156
N1—H1*N*1···N4^v^	0.97 (2)	2.29 (2)	3.218 (2)	161 (2)
N1—H1*N*1···N2	0.97 (2)	2.42 (2)	2.928 (2)	112 (2)
N1—H2*N*1···Cl1^vi^	0.97 (2)	2.08 (2)	3.034 (2)	168 (2)
N1—H3*N*1···Cl1^iii^	0.93 (2)	2.27 (2)	3.188 (2)	167 (2)

Symmetry codes: (i) −*x*+1, −*y*+1, −*z*+1; (ii) *x*−1, −*y*+1/2, *z*−1/2; (iii) *x*, *y*−1, *z*; (iv) −*x*+2, *y*+1/2, −*z*+3/2; (v) −*x*+1, −*y*, −*z*+1; (vi) −*x*+1, *y*−1/2, −*z*+1/2.

### 3,6,6-Trimethyl-4-oxo-1-(pyridin-2-yl)-4,5,6,7-tetrahydro-1H-indazol-7-aminium chloride monohydrate (2) Crystal data

**Table:**

C_15_H_19_N_4_O^+^·Cl^−^·H_2_O	*F*(000) = 688
*M**_r_* = 324.81	*D*_x_ = 1.382 Mg m^−^^3^
Monoclinic, *P*2_1_/*c*	Mo *K*α radiation, λ = 0.71073 Å
*a* = 10.1855 (2) Å	Cell parameters from 8626 reflections
*b* = 7.4951 (2) Å	θ = 1.0–27.5°
*c* = 20.7961 (4) Å	µ = 0.26 mm^−^^1^
β = 100.545 (1)°	*T* = 190 K
*V* = 1560.79 (6) Å^3^	Block, colourless
*Z* = 4	0.42 × 0.25 × 0.14 mm

### 3,6,6-Trimethyl-4-oxo-1-(pyridin-2-yl)-4,5,6,7-tetrahydro-1H-indazol-7-aminium chloride monohydrate (2) Data collection

**Table:**

Nonius KappaCCD diffractometer	*R*_int_ = 0.023
Radiation source: fine-focus sealed tube	θ_max_ = 27.7°, θ_min_ = 3.6°
CCD scans	*h* = −13→13
5549 measured reflections	*k* = −9→9
3552 independent reflections	*l* = −27→26
2874 reflections with *I* > 2σ(*I*)	

### 3,6,6-Trimethyl-4-oxo-1-(pyridin-2-yl)-4,5,6,7-tetrahydro-1H-indazol-7-aminium chloride monohydrate (2) Refinement

**Table:**

Refinement on *F*^2^	0 restraints
Least-squares matrix: full	Hydrogen site location: mixed
*R*[*F*^2^ > 2σ(*F*^2^)] = 0.039	H atoms treated by a mixture of independent and constrained refinement
*wR*(*F*^2^) = 0.102	*w* = 1/[σ^2^(*F*_o_^2^) + (0.0426*P*)^2^ + 0.794*P*] where *P* = (*F*_o_^2^ + 2*F*_c_^2^)/3
*S* = 1.06	(Δ/σ)_max_ < 0.001
3552 reflections	Δρ_max_ = 0.29 e Å^−^^3^
222 parameters	Δρ_min_ = −0.28 e Å^−^^3^

### 3,6,6-Trimethyl-4-oxo-1-(pyridin-2-yl)-4,5,6,7-tetrahydro-1H-indazol-7-aminium chloride monohydrate (2) Special details

**Table:**

Geometry. All esds (except the esd in the dihedral angle between two l.s. planes) are estimated using the full covariance matrix. The cell esds are taken into account individually in the estimation of esds in distances, angles and torsion angles; correlations between esds in cell parameters are only used when they are defined by crystal symmetry. An approximate (isotropic) treatment of cell esds is used for estimating esds involving l.s. planes.

### 3,6,6-Trimethyl-4-oxo-1-(pyridin-2-yl)-4,5,6,7-tetrahydro-1H-indazol-7-aminium chloride monohydrate (2) Fractional atomic coordinates and isotropic or equivalent isotropic displacement parameters (Å^2^)

**Table:**

	*x*	*y*	*z*	*U*_iso_*/*U*_eq_	
Cl1	0.06154 (4)	0.62526 (6)	0.39636 (2)	0.02975 (13)	
O1	0.45919 (12)	−0.12171 (17)	0.26173 (5)	0.0286 (3)	
N2	0.33632 (13)	0.28400 (18)	0.51823 (6)	0.0210 (3)	
O1W	−0.00940 (13)	0.25508 (19)	0.46352 (7)	0.0328 (3)	
N3	0.48296 (12)	0.17260 (17)	0.45521 (6)	0.0159 (3)	
N1	0.18153 (14)	0.01688 (19)	0.43612 (7)	0.0183 (3)	
N4	0.61172 (12)	0.13967 (18)	0.44629 (6)	0.0196 (3)	
C5	0.24311 (14)	0.1376 (2)	0.39147 (7)	0.0155 (3)	
H5	0.223932	0.261303	0.401963	0.019*	
C12	0.57141 (16)	0.3394 (2)	0.55440 (8)	0.0219 (3)	
H12	0.658465	0.326736	0.547312	0.026*	
C11	0.46319 (15)	0.2686 (2)	0.51150 (7)	0.0174 (3)	
C4	0.39179 (14)	0.1150 (2)	0.40322 (7)	0.0153 (3)	
C6	0.18454 (15)	0.1060 (2)	0.31807 (7)	0.0176 (3)	
C10	0.03220 (16)	0.0870 (2)	0.30801 (8)	0.0246 (4)	
H10A	0.009876	−0.020414	0.328751	0.037*	
H10B	−0.003916	0.081667	0.262056	0.037*	
H10C	−0.004705	0.187831	0.326935	0.037*	
C9	0.21584 (16)	0.2703 (2)	0.27933 (8)	0.0227 (3)	
H9A	0.180810	0.375246	0.296617	0.034*	
H9B	0.175456	0.256460	0.234144	0.034*	
H9C	0.310786	0.282002	0.283039	0.034*	
C3	0.46350 (15)	0.0404 (2)	0.35966 (7)	0.0166 (3)	
C8	0.39804 (16)	−0.0515 (2)	0.30028 (7)	0.0192 (3)	
C15	0.31230 (18)	0.3783 (2)	0.56960 (8)	0.0270 (4)	
H15	0.224058	0.395222	0.574240	0.032*	
C7	0.24693 (16)	−0.0608 (2)	0.29224 (7)	0.0213 (3)	
H7A	0.223032	−0.164981	0.315275	0.026*	
H7B	0.209284	−0.075806	0.246229	0.026*	
C14	0.4120 (2)	0.4514 (2)	0.61590 (8)	0.0299 (4)	
H14	0.391561	0.513786	0.651450	0.036*	
C13	0.54279 (19)	0.4297 (2)	0.60821 (8)	0.0280 (4)	
H13	0.611825	0.475897	0.639260	0.034*	
C2	0.59994 (15)	0.0631 (2)	0.38810 (7)	0.0196 (3)	
C1	0.72083 (17)	0.0203 (3)	0.36008 (9)	0.0295 (4)	
H1A	0.799516	0.046429	0.391767	0.044*	
H1B	0.720784	0.090854	0.321550	0.044*	
H1C	0.720059	−0.104005	0.348878	0.044*	
H1W	−0.026 (3)	0.288 (4)	0.4977 (14)	0.063 (9)*	
H2W	0.012 (3)	0.362 (4)	0.4440 (13)	0.066 (8)*	
H1N1	0.1557 (19)	−0.083 (3)	0.4188 (9)	0.024 (5)*	
H2N1	0.244 (2)	−0.009 (3)	0.4766 (11)	0.036 (5)*	
H3N1	0.112 (2)	0.080 (3)	0.4513 (11)	0.049 (7)*	

### 3,6,6-Trimethyl-4-oxo-1-(pyridin-2-yl)-4,5,6,7-tetrahydro-1H-indazol-7-aminium chloride monohydrate (2) Atomic displacement parameters (Å^2^)

**Table:**

	*U*^11^	*U*^22^	*U*^33^	*U*^12^	*U*^13^	*U*^23^
Cl1	0.0343 (2)	0.0261 (2)	0.0301 (2)	−0.00602 (18)	0.00909 (17)	−0.00145 (17)
O1	0.0328 (7)	0.0360 (7)	0.0182 (6)	0.0076 (5)	0.0077 (5)	−0.0040 (5)
N2	0.0237 (7)	0.0214 (7)	0.0185 (6)	0.0011 (6)	0.0053 (5)	−0.0027 (6)
O1W	0.0343 (7)	0.0287 (7)	0.0385 (8)	0.0014 (6)	0.0152 (6)	−0.0051 (6)
N3	0.0144 (6)	0.0174 (6)	0.0156 (6)	−0.0002 (5)	0.0018 (5)	0.0000 (5)
N1	0.0185 (7)	0.0208 (7)	0.0162 (6)	−0.0037 (6)	0.0045 (5)	−0.0014 (6)
N4	0.0140 (6)	0.0226 (7)	0.0222 (7)	0.0005 (5)	0.0036 (5)	0.0019 (6)
C5	0.0155 (7)	0.0168 (7)	0.0143 (7)	−0.0016 (6)	0.0026 (5)	0.0007 (6)
C12	0.0250 (8)	0.0171 (7)	0.0211 (8)	−0.0021 (6)	−0.0029 (6)	0.0022 (6)
C11	0.0226 (8)	0.0141 (7)	0.0147 (7)	−0.0004 (6)	0.0018 (6)	0.0019 (6)
C4	0.0164 (7)	0.0153 (7)	0.0138 (7)	−0.0017 (6)	0.0022 (5)	0.0020 (6)
C6	0.0156 (7)	0.0224 (8)	0.0140 (7)	−0.0015 (6)	0.0003 (5)	0.0008 (6)
C10	0.0177 (8)	0.0332 (9)	0.0216 (8)	−0.0044 (7)	−0.0002 (6)	−0.0003 (7)
C9	0.0221 (8)	0.0251 (9)	0.0193 (7)	−0.0006 (7)	−0.0004 (6)	0.0054 (7)
C3	0.0185 (7)	0.0171 (7)	0.0146 (7)	0.0000 (6)	0.0039 (6)	0.0017 (6)
C8	0.0262 (8)	0.0180 (7)	0.0139 (7)	0.0015 (6)	0.0045 (6)	0.0031 (6)
C15	0.0344 (9)	0.0263 (9)	0.0222 (8)	0.0030 (7)	0.0100 (7)	−0.0024 (7)
C7	0.0244 (8)	0.0229 (8)	0.0163 (7)	−0.0042 (7)	0.0033 (6)	−0.0034 (6)
C14	0.0506 (12)	0.0225 (9)	0.0173 (7)	−0.0006 (8)	0.0079 (7)	−0.0035 (7)
C13	0.0427 (11)	0.0181 (8)	0.0189 (8)	−0.0055 (7)	−0.0061 (7)	−0.0014 (7)
C2	0.0183 (8)	0.0210 (8)	0.0201 (7)	0.0016 (6)	0.0054 (6)	0.0036 (6)
C1	0.0205 (8)	0.0392 (11)	0.0310 (9)	0.0027 (8)	0.0109 (7)	0.0000 (8)

### 3,6,6-Trimethyl-4-oxo-1-(pyridin-2-yl)-4,5,6,7-tetrahydro-1H-indazol-7-aminium chloride monohydrate (2) Geometric parameters (Å, º)

**Table:**

O1—C8	1.2207 (19)	C6—C7	1.543 (2)
N2—C11	1.330 (2)	C10—H10A	0.9600
N2—C15	1.340 (2)	C10—H10B	0.9600
O1W—H1W	0.80 (3)	C10—H10C	0.9600
O1W—H2W	0.94 (3)	C9—H9A	0.9600
N3—C4	1.3601 (18)	C9—H9B	0.9600
N3—N4	1.3800 (17)	C9—H9C	0.9600
N3—C11	1.4196 (19)	C3—C2	1.418 (2)
N1—C5	1.5127 (19)	C3—C8	1.464 (2)
N1—H1N1	0.85 (2)	C8—C7	1.519 (2)
N1—H2N1	0.98 (2)	C15—C14	1.378 (3)
N1—H3N1	0.95 (2)	C15—H15	0.9300
N4—C2	1.325 (2)	C7—H7A	0.9700
C5—C4	1.499 (2)	C7—H7B	0.9700
C5—C6	1.5522 (19)	C14—C13	1.380 (3)
C5—H5	0.9800	C14—H14	0.9300
C12—C13	1.384 (2)	C13—H13	0.9300
C12—C11	1.390 (2)	C2—C1	1.491 (2)
C12—H12	0.9300	C1—H1A	0.9600
C4—C3	1.382 (2)	C1—H1B	0.9600
C6—C10	1.534 (2)	C1—H1C	0.9600
C6—C9	1.537 (2)		
			
C11—N2—C15	116.89 (14)	H10B—C10—H10C	109.5
H1W—O1W—H2W	103 (3)	C6—C9—H9A	109.5
C4—N3—N4	111.34 (12)	C6—C9—H9B	109.5
C4—N3—C11	129.57 (13)	H9A—C9—H9B	109.5
N4—N3—C11	118.90 (12)	C6—C9—H9C	109.5
C5—N1—H1N1	113.5 (13)	H9A—C9—H9C	109.5
C5—N1—H2N1	111.4 (12)	H9B—C9—H9C	109.5
H1N1—N1—H2N1	106.9 (18)	C4—C3—C2	105.85 (13)
C5—N1—H3N1	108.9 (15)	C4—C3—C8	122.00 (13)
H1N1—N1—H3N1	112.7 (19)	C2—C3—C8	132.00 (14)
H2N1—N1—H3N1	102.9 (18)	O1—C8—C3	123.26 (15)
C2—N4—N3	105.71 (12)	O1—C8—C7	122.43 (14)
C4—C5—N1	110.64 (12)	C3—C8—C7	114.23 (13)
C4—C5—C6	109.76 (12)	N2—C15—C14	123.21 (17)
N1—C5—C6	112.59 (12)	N2—C15—H15	118.4
C4—C5—H5	107.9	C14—C15—H15	118.4
N1—C5—H5	107.9	C8—C7—C6	113.45 (13)
C6—C5—H5	107.9	C8—C7—H7A	108.9
C13—C12—C11	116.49 (16)	C6—C7—H7A	108.9
C13—C12—H12	121.8	C8—C7—H7B	108.9
C11—C12—H12	121.8	C6—C7—H7B	108.9
N2—C11—C12	124.81 (14)	H7A—C7—H7B	107.7
N2—C11—N3	114.70 (13)	C15—C14—C13	118.40 (16)
C12—C11—N3	120.48 (14)	C15—C14—H14	120.8
N3—C4—C3	106.48 (13)	C13—C14—H14	120.8
N3—C4—C5	128.05 (13)	C14—C13—C12	120.12 (16)
C3—C4—C5	125.34 (13)	C14—C13—H13	119.9
C10—C6—C9	107.73 (13)	C12—C13—H13	119.9
C10—C6—C7	110.38 (13)	N4—C2—C3	110.57 (13)
C9—C6—C7	109.20 (12)	N4—C2—C1	120.48 (14)
C10—C6—C5	110.21 (12)	C3—C2—C1	128.90 (15)
C9—C6—C5	108.27 (12)	C2—C1—H1A	109.5
C7—C6—C5	110.96 (12)	C2—C1—H1B	109.5
C6—C10—H10A	109.5	H1A—C1—H1B	109.5
C6—C10—H10B	109.5	C2—C1—H1C	109.5
H10A—C10—H10B	109.5	H1A—C1—H1C	109.5
C6—C10—H10C	109.5	H1B—C1—H1C	109.5
H10A—C10—H10C	109.5		
			
C4—N3—N4—C2	0.70 (17)	N3—C4—C3—C2	−1.90 (16)
C11—N3—N4—C2	−174.73 (13)	C5—C4—C3—C2	174.12 (14)
C15—N2—C11—C12	1.5 (2)	N3—C4—C3—C8	174.09 (13)
C15—N2—C11—N3	−178.02 (14)	C5—C4—C3—C8	−9.9 (2)
C13—C12—C11—N2	1.0 (2)	C4—C3—C8—O1	−178.45 (15)
C13—C12—C11—N3	−179.50 (14)	C2—C3—C8—O1	−3.6 (3)
C4—N3—C11—N2	9.7 (2)	C4—C3—C8—C7	−1.5 (2)
N4—N3—C11—N2	−175.82 (13)	C2—C3—C8—C7	173.30 (16)
C4—N3—C11—C12	−169.86 (15)	C11—N2—C15—C14	−2.8 (2)
N4—N3—C11—C12	4.6 (2)	O1—C8—C7—C6	−148.01 (15)
N4—N3—C4—C3	0.81 (17)	C3—C8—C7—C6	35.01 (18)
C11—N3—C4—C3	175.62 (14)	C10—C6—C7—C8	179.76 (13)
N4—N3—C4—C5	−175.06 (14)	C9—C6—C7—C8	61.51 (16)
C11—N3—C4—C5	−0.3 (2)	C5—C6—C7—C8	−57.76 (16)
N1—C5—C4—N3	−72.98 (19)	N2—C15—C14—C13	1.5 (3)
C6—C5—C4—N3	162.17 (14)	C15—C14—C13—C12	1.2 (3)
N1—C5—C4—C3	111.88 (16)	C11—C12—C13—C14	−2.3 (2)
C6—C5—C4—C3	−13.0 (2)	N3—N4—C2—C3	−1.93 (17)
C4—C5—C6—C10	167.33 (13)	N3—N4—C2—C1	175.62 (15)
N1—C5—C6—C10	43.61 (17)	C4—C3—C2—N4	2.45 (18)
C4—C5—C6—C9	−75.07 (15)	C8—C3—C2—N4	−172.97 (15)
N1—C5—C6—C9	161.21 (13)	C4—C3—C2—C1	−174.84 (17)
C4—C5—C6—C7	44.75 (16)	C8—C3—C2—C1	9.7 (3)
N1—C5—C6—C7	−78.96 (15)		

### 3,6,6-Trimethyl-4-oxo-1-(pyridin-2-yl)-4,5,6,7-tetrahydro-1H-indazol-7-aminium chloride monohydrate (2) Hydrogen-bond geometry (Å, º)

**Table:**

*D*—H···*A*	*D*—H	H···*A*	*D*···*A*	*D*—H···*A*
C12—H12···Cl1^i^	0.93	2.90	3.7006 (17)	145
C14—H14···O1^ii^	0.93	2.41	3.244 (2)	149
O1*W*—H1*W*···Cl1^iii^	0.80 (3)	2.39 (3)	3.185 (2)	176 (3)
O1*W*—H2*W*···Cl1	0.94 (3)	2.31 (3)	3.247 (2)	179 (2)
N1—H1*N*1···Cl1^iv^	0.85 (2)	2.40 (2)	3.228 (2)	165 (2)
N1—H2*N*1···N4^v^	0.98 (2)	2.20 (2)	3.1475 (19)	164 (2)
N1—H3*N*1···O1*W*	0.95 (2)	1.85 (3)	2.775 (2)	162 (2)

Symmetry codes: (i) −*x*+1, −*y*+1, −*z*+1; (ii) *x*, −*y*+1/2, *z*+1/2; (iii) −*x*, −*y*+1, −*z*+1; (iv) *x*, *y*−1, *z*; (v) −*x*+1, −*y*, −*z*+1.

## References

[bb1] Abdel-Rahman, H. M. & Ozadali, K. (2012). *Arch. Pharm. Pharm. Med. Chem.* **345**, 878–883. 10.1002/ardp.201200193 22907715

[bb2] Ansari, A., Ali, A., Asif, M. & Shamsuzzaman (2017). *New J. Chem.* **41**, 16–41.

[bb3] Bassyouni, F., El Din Gaffer, A., Roaiah, H., El-Senousy, W. M., El Nakkady, S. S. & &Rehim, M. A. (2016). *Res. J. Pharm. Biol. Chem. Sci*, **7**, 24–37.

[bb33] Bruker (20014). *COLLECT*. Bruker AXS Inc., Madison, Wisconsin, USA.

[bb4] Burch, J. D., Barrett, K., Chen, Y., DeVoss, J., Eigenbrot, C., Goldsmith, R., Ismaili, M. H. A., Lau, K., Lin, Z., Ortwine, D. F., Zarrin, A. A., McEwan, P. A., Barker, J. J., Ellebrandt, C., Kordt, D., Stein, D. B., Wang, X., Chen, Y., Hu, B., Xu, X., Yuen, P.-W., Zhang, Y. & Pei, Z. (2015). *J. Med. Chem.* **58**, 3806–3816. 10.1021/jm501998m 25844760

[bb5] Burla, M. C., Caliandro, R., Camalli, M., Carrozzini, B., Cascarano, G. L., De Caro, L., Giacovazzo, C., Polidori, G. & Spagna, R. (2005). *J. Appl. Cryst.* **38**, 381–388.

[bb6] Claramunt, R. M., López, C., Pérez-Medina, C., Pinilla, E., Torres, M. R. & Elguero, J. (2006). *Tetrahedron*, **62**, 11704–11713.

[bb7] Etter, M. C. (1990). *Acc. Chem. Res.* **23**, 120–126.

[bb8] Faidallah, H. M., Khan, K. A., Rostom, S. A. F. & Asiri, A. M. (2013). *J. Enzyme Inhib. Med. Chem.* **28**, 495–508. 10.3109/14756366.2011.653354 22329488

[bb9] Farrugia, L. J. (2012). *J. Appl. Cryst.* **45**, 849–854.

[bb10] Gaikwad, D. D., Chapolikar, A. D., Devkate, C. G., Warad, K. D., Tayade, A. P., Pawar, R. P. & Domb, A. J. (2015). *Eur. J. Med. Chem.* **90**, 707–731. 10.1016/j.ejmech.2014.11.029 25506810

[bb11] Groom, C. R., Bruno, I. J., Lightfoot, M. P. & Ward, S. C. (2016). *Acta Cryst.* B**72**, 171–179. 10.1107/S2052520616003954 PMC4822653 27048719

[bb12] Guo, S., Song, Y., Huang, Q., Yuan, H., Wan, B., Wang, Y., He, R., Beconi, M. G., Franzblau, S. G. & Kozikowski, A. P. (2010). *J. Med. Chem.* **53**, 649–659. 10.1021/jm901235p 20000470

[bb13] Heifetz, A., Trani, G., Aldeghi, M., MacKinnon, C. H., McEwan, P. A., Brookfield, F. A., Chudyk, E. I., Bodkin, M., Pei, Z., Burch, J. D. & Ortwine, D. F. (2016). *J. Med. Chem.* **59**, 4352–4363. 10.1021/acs.jmedchem.6b00045 26950250

[bb14] Jia, J., Xu, Q.-C., Li, R.-C., Tang, X., He, Y.-F., Zhang, M.-Y., Zhang, Y. & Xing, G.-W. (2012). *Org. Biomol. Chem.* **10**, 6279–6286. 10.1039/c2ob25852h 22722482

[bb15] Khlebnicova, T. S., Piven, Y. A., Baranovsky, A. V., Lakhvich, F. A., Shishkina, S. V., Zicāne, D., Tetere, Z., Rāviņa, I., Kumpiņš, V., Rijkure, I., Mieriņa, I., Peipiņš, U. & Turks, M. (2017). *Steroids*, **117**, 77–89. 10.1016/j.steroids.2016.08.002 27500691

[bb16] Khlebnikova, T. S., Piven’, Y. A., Baranovskii, A. V. & Lakhvich, F. A. (2012). *Russ. J. Org. Chem.* **48**, 411–418.

[bb17] Kim, J., Song, H. & Park, S. B. (2010). *Eur. J. Org. Chem.* pp. 3815–3822.

[bb18] Matthews, J. M., McNally, J. J., Connolly, P. J., Xia, M., Zhu, B., Black, S., Chen, C., Hou, C., Liang, Y., Tang, Y. & Macielag, M. J. (2016). *Bioorg. Med. Chem. Lett.* **26**, 5346–5349. 10.1016/j.bmcl.2016.09.025 27671496

[bb19] Murugavel, K., Amirthaganesan, S. & R. T. S. (2010). *Chem. Heterocycl. C.* **46**, 302–306.

[bb20] Nakhai, A. & Bergman, J. (2009). *Tetrahedron*, **65**, 2298–2306.

[bb21] Otwinowski, Z. & Minor, W. (1997). *Methods in Enzymology*, Vol. 276, *Macromolecular Crystallography*, Part A, edited by C. W. Carter Jr & R. M. Sweet, pp. 307–326. New York: Academic Press.

[bb22] Polo, E., Trilleras, J., Ramos, J., Galdámez, A., Quiroga, J. & Gutierrez, M. (2016). *Molecules*, **21**, article number 903. 10.3390/molecules21070903 PMC6274391 27409599

[bb23] Scala, A., Piperno, A., Risitano, F., Cirmi, S., Navarra, M. & Grassi, G. (2015). *Mol. Divers.* **19**, 473–480. 10.1007/s11030-015-9583-5 25784276

[bb24] Sheldrick, G. M. (2015). *Acta Cryst* C**71**, 3–8.

[bb25] Silva, V. L. M., Silva, A. M. S., Pinto, D. C. G. A. & Cavaleiro, J. (2006). *Synlett*, pp. 1369–1373.

[bb26] Song, H., Lee, H., Kim, J. & Park, S. B. (2012). *ACS Comb. Sci.* **14**, 66–74. 10.1021/co200150d 22107604

[bb27] Strakova, I., Kumpiņa, I., Rjabovs, V., Lugiņina, J., Belyakov, S. & Turks, M. (2011). *Tetrahedron Asymmetry*, **22**, 728–739.

[bb28] Strakova, I., Turks, M. & Strakovs, A. (2009). *Tetrahedron Lett.* **50**, 3046–3049.

[bb29] Turks, M., Strakova, I., Gorovojs, K., Belyakov, S., Piven, Y. A., Khlebnicova, T. S. & Lakhvich, F. A. (2012). *Tetrahedron*, **68**, 6131–6140.

[bb30] Westrip, S. P. (2010). *J. Appl. Cryst.* **43**, 920–925.

[bb31] Wiener, J. J. M., Gomez, L., Venkatesan, H., Santillán, A. Jr, Allison, B. D., Schwarz, K. L., Shinde, S., Tang, L., Hack, M. D., Morrow, B. J., Motley, S. T., Goldschmidt, R. M., Shaw, K. J., Jones, T. K. & Grice, C. A. (2007). *Bioorg. Med. Chem. Lett.* **17**, 2718–2722. 10.1016/j.bmcl.2007.03.004 17382544

[bb32] Wu, Z.-W., Song, S.-Y., Li, L., Lu, H.-L., Lieberman, B., Huang, Y.-S. & Mach, R. H. (2015). *Bioorg. Med. Chem.* **23**, 1463–1471. 10.1016/j.bmc.2015.02.012 25752422

